# Neuromedin U uses Gα_i2_ and Gα_o_ to suppress glucose-stimulated Ca^2+^ signaling and insulin secretion in pancreatic β cells

**DOI:** 10.1371/journal.pone.0250232

**Published:** 2021-04-15

**Authors:** Weidong Zhang, Hideyuki Sakoda, Yuki Nakazato, Md Nurul Islam, François Pattou, Julie Kerr-Conte, Masamitsu Nakazato

**Affiliations:** 1 Division of Neurology, Respirology, Endocrinology and Metabolism, Department of Internal Medicine, Faculty of Medicine, University of Miyazaki, Miyazaki, Japan; 2 UNIV. LILLE, INSERM, CHU LILLE, U1190, Translational Research Laboratory for Diabetes -European Genomics Institute for Diabetes, Lille, France; 3 AMED-CREST, Agency for Medical Research and Development, Tokyo, Japan; Medical University of Vienna, AUSTRIA

## Abstract

Neuromedin U (NMU), a highly conserved peptide in mammals, is involved in a wide variety of physiological processes, including impairment of pancreatic β-cell function via induction of mitochondrial dysfunction and endoplasmic reticulum (ER) stress, ultimately suppressing insulin secretion. NMU has two receptors, NMU receptor 1 (NMUR1) and NMUR2, both of which are G-protein–coupled receptors (GPCRs). Only NMUR1 is expressed in mouse islets and β cell–derived MIN6-K8 cells. The molecular mechanisms underlying the insulinostatic action mediated by NMUR1 in β cells have yet to be elucidated. In this study, we explored the molecular mechanism driving impairment of insulin secretion in β cells by the NMU–NMUR1 axis. Pretreatment with the Gα_i/o_ inhibitor *Bordetella pertussis* toxin (PTX), but not the Gα_q_ inhibitor YM254890, abolished NMU-induced suppression of glucose-stimulated insulin secretion and calcium response in β cells. Knockdown of Gα_i2_ and Gα_o_ in β cells counteracted NMU-induced suppression of insulin secretion and gene alterations related to mitochondrial fusion (*Mfn1*, *Mfn2*), fission (*Fis1*, *Drp1*), mitophagy (*Pink1*, *Park2*), mitochondrial dynamics (*Pgc-1α*, *Nrf1*, and *Tfam*), ER stress (*Chop*, *Atp2a3*, *Ryr2*, and *Itpr2*), intracellular ATP level, and mitochondrial membrane potential. NMU decreased forskolin-stimulated intracellular cAMP in both mouse and human islets. We concluded that NMUR1 coupled to PTX-sensitive Gα_i2_ and Gα_o_ proteins in β cells reduced intracellular Ca^2+^ influx and cAMP level, thereby causing β-cell dysfunction and impairment. These results highlight a novel signaling mechanism of NMU and provide valuable insights into the further investigation of NMU functions in β-cell biology.

## Introduction

Pancreatic β cells, the predominant cell type within the pancreatic islets of mammals, are the sole source of circulating insulin. Beta-cell function and maintenance are regulated by a complex set of interacting factors and involve autocrine, paracrine, and humoral signaling [1–3]. The regulatory network that controls glucose-stimulated insulin secretion (GSIS) is multifactorial and complicated. A number of G-protein–coupled receptors (GPCRs) and peptides produced in β cells participate in the regulation of insulin secretion and islet homeostasis [4]. Beta cell–derived peptides such as amylin, cholecystokinin, gastrin, oxytocin, and xenin enhance GSIS, whereas urocortin-3 suppresses it [3, 5].

Neuromedin U (NMU) is a 23–25-amino acid peptide isolated from the spinal cord, named for its contractile effect on the rat uterus [6, 7]. NMU has emerged as a new player in the regulation of appetite control and energy and glucose homeostasis [8–10]. NMU has two cognate GPCRs: NMU receptor 1 (NMUR1), which is expressed predominantly in peripheral tissues, and NMUR2, which is expressed abundantly in the central nervous system [9, 10]. NMU and NMUR1 are expressed in pancreatic islets of mice, rats, and humans, whereas NMUR2 is not [11–14]. NMU produced in β cells suppresses insulin secretion and causes β-cell failure by inducing mitochondrial dysfunction and endoplasmic reticulum (ER) stress [11, 14, 15]. These observations suggest that NMU directly regulates β-cell function and maintenance via NMUR1.

GPCRs propagate signals in cells through heterotrimeric G-proteins, which consist of α-, β-, and γ-subunits. Upon ligand binding, GPCRs and heterotrimeric G-proteins change their conformation and transduce signals inside cells, linking membrane receptor activation to intracellular effectors [16]. Gα, the major determinant of GPCR specificity, has four subfamilies: Gα_s_, Gα_q_, Gα_12/13_, and Gα_i/o_ [17, 18]. All types of Gα proteins are expressed in β cells and are involved in the regulation of insulin secretion [19]. For normal β cells, somatostatin and ghrelin activate Gα_i/o_ proteins to inhibit adenosine cAMP production, thereby suppressing insulin secretion [20, 21]; Glucagon-like peptide-1 activates Gα_s_ to stimulate adenosine cAMP production, and cholecystokinin activates Gα_q_ to increase intracellular Ca^2+^ influx; both cAMP and Ca^2+^ stimulate insulin secretion [22, 23].

NMUR1 mainly conducts Gα_q_ signals, whereas NMUR2 mainly acts in the Gα_i_ pathway. These findings were obtained using non–β-cell lines with ectopic overexpression of NMUR1 or NMUR2 [24–26]. Early work revealed that GPCRs are promiscuous in terms of G-protein coupling [27]. Several studies revealed that individual GPCRs can bind to different G-proteins in different tissues. For example, in β cells, the Class B GPCR glucagon receptor binds to both Gα_s_ and Gα_q_ proteins [4], but also binds to Gα_i/o_ to regulate calcium signaling in HEK293 cells and mediate cAMP accumulation in canine hepatocytes [28, 29]. In mouse dorsal root ganglia neurons, NMUR1 binds to Gα_i/o_ to inhibit the T-type Ca^2+^ channel current via the PKA signaling pathway, whereas in mouse hippocampal neurons this interaction inhibits the L-type Ca^2+^ channel current via the PI3K–PKC signaling pathway [30, 31]. Given that NMUR1, but not NMUR2, is expressed in β cells, and NMU suppresses intracellular calcium mobilization and insulin secretion, we hypothesized that NMUR1 might use Gα_i/o_ for signal transduction in β cells. Using β cell‒derived MIN6-K8 cells, isolated mouse and human islets, and single islet cells, we identified the molecular mechanisms by which NMU and NMUR1 regulate insulin secretion in β cells.

## Materials and methods

### Animals, cell culture, and islet isolation

C57BL/6 J wild-type mice (9-week-old male, Charles River Laboratories Japan, Yokohama, Japan) were maintained in individual cages under a 12-h/12-h light–dark cycle (light on 08:00–20:00) at controlled temperatures (21–23°C). Animals were fed standard rodent chow pellets with water *ad libitum*. All animal experiments were approved by the Animal Care and Use Committee of University of Miyazaki and complied with the guidelines of Japanese Physiological Society for the care and use of laboratory animals at University of Miyazaki.

MIN6-K8 cells [32] were cultured at 37 °C under 5% CO_2_ in Dulbecco’s modified Eagle’s medium (DMEM) supplemented with 10% heat-inactivated fetal bovine serum (FBS) (Thermo Fisher Scientific, Waltham, MA, USA; Cat. No.10270-106) and 1% penicillin–streptomycin (Wako, Osaka, Japan, cat No: 168–23191).

Pancreatic islets were isolated from C57BL/6J mice by a modified collagenase digestion method [33]. A combination anesthetic was prepared, consisting of 0.3 mg/kg of medetomidine, 4.0 mg/kg of midazolam, and 5.0 mg/kg of butorphanol, and administered to mice by intraperitoneal injection. After anesthesia and sacrifice, the pancreas was cut into small pieces by mincing with scissors for approximately 1 min. The minced tissue was incubated in 10 ml Collagenase P (1 mg/ml in HBSS, Roche Diagnostics) for 15 min in a 37 °C water bath. After addition of 30 ml of cold HBSS, the tube was shaken by hand for 60 s until it appeared homogeneous. The sample was centrifuged for 2 min at 112 rcf, and the pellet was washed twice with cold HBSS. The pellet was resuspended in RPMI supplemented with 10% FBS and 1% penicillin–streptomycin. Isolated pancreatic islets were picked under a stereomicroscope using an Eppendorf pipette. Before experiments were performed, islets were incubated overnight at 37 °C under 5% CO_2_ in RPMI or dispersed into single cells by 10-min incubation at 37 °C with 0.25% trypsin. Dispersed islet cells were incubated in RPMI for up to 2 days at 37 °C before use.

Human pancreatic islets isolated from cadaveric heart beating donors as previously described [34], after next-of-kin consent, were kindly provided by the University Hospital of Lille via the European Consortium for Islet Transplantation (ECIT) human islet distribution program supported by the Juvenile Diabetes Research Foundation (JDRF). The use of human specimens for research and exportation of human islets was granted respectively by the French Ministry of Higher Education and Research and the Agence de la biomédecine. Islet purity was assessed as the percentage of endocrine clusters positive to dithizone staining (range: 80–90%). Human pancreata were harvested from adult brain-deceased donors in agreement with the French Regulations and with our Institutional Ethical Committee (« Comité d’Ethique du Centre Hospitialier Régional et Universitaire de Lille »). France has presumed consent legislation in place for deceased donors. Human islet preparations insufficient in number for clinical transplantation can be used for research when consent for RESEARCH donation has been given (NOTE: Informed consent from the next of kin, is obtained on the behalf of the deceased by the National French Procurement Agency «Agence de la biomédecine». Consent was verbal. In France it is presumed that you agree to organ donation unless you have expressed your refusal in the «RNR: Registre National de Refus » (National Registry for Refusal of organ donation). The Agence de la biomédecine Coordinators ask for verbal consent and then consult the RNR (ie written refusal). Therefore the written refusal is based on the RNR (Registre National de Refus: National Registry for Refusal of organ donation). Documentation of the process: The Agence de la biomédecine has standardized procedure applicable nationally for Coordinators to assure that the informed consent procedure is identical throughout France.). No minors were used.

### In vitro insulin secretion assay

**Fig 1:**
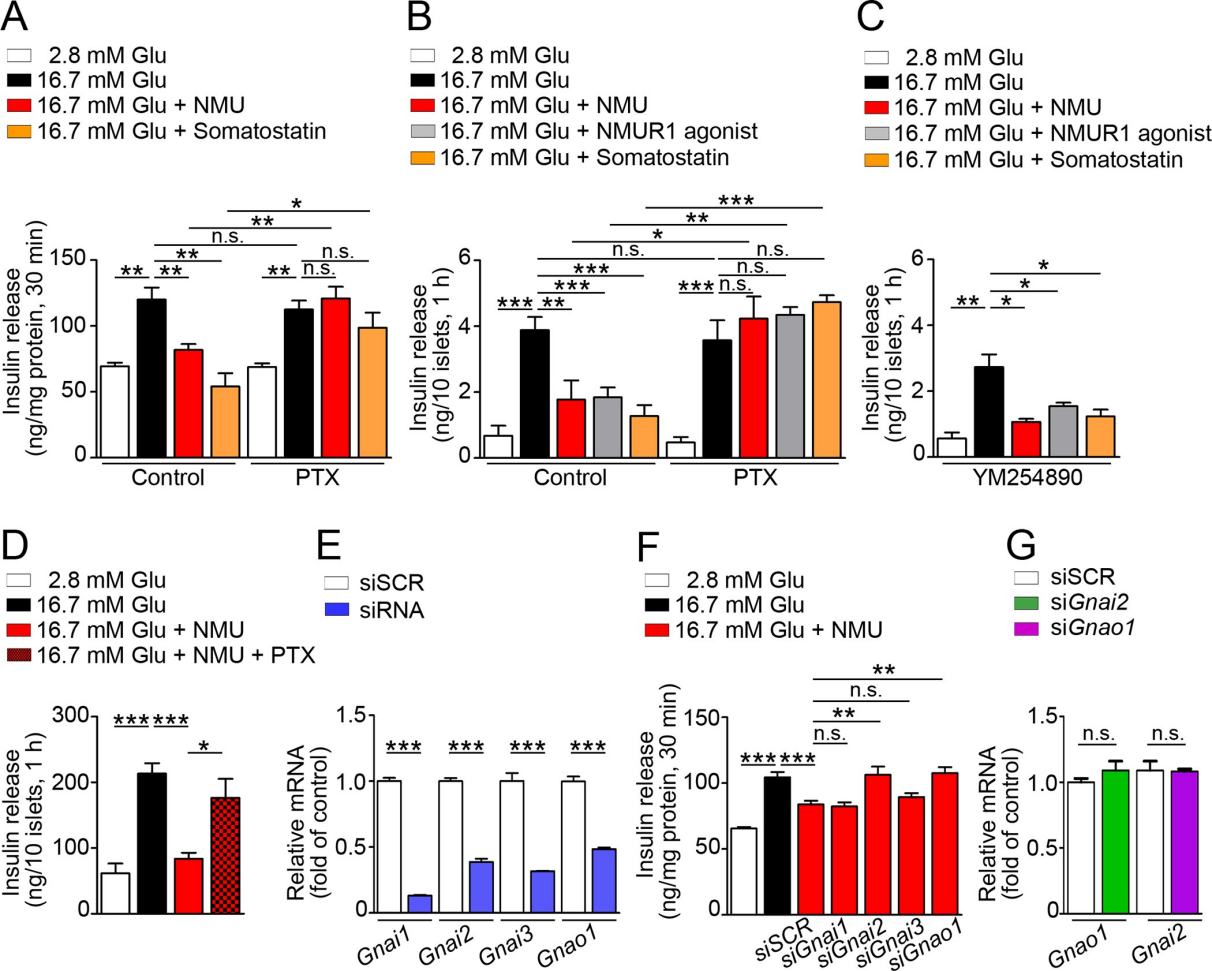
Involvement of Gα_i/o_ signaling in NMU-suppressed GSIS. Insulin secretion from MIN6-K8 cells (A) (*n* = 4), isolated mouse islets (B, C) (*n* = 4), and human islets (D) (*n* = 4) with or without PTX or YM254890 pretreatment. (E) Efficacy of Gα_i/o_ knockdown in MIN6-K8 cells (n = 4). (F) NMU-induced suppression of GSIS after Gα_i/o_ knockdown in MIN6-K8 cells (n = 4). (G) mRNA levels of *Gnao1* or *Gnai2* in MIN6-K8 cells after respective knockdown of *Gnai2* or *Gnao1* (n = 4). *P < 0.05, **P < 0.01, ***P < 0.001. n.s., not significant.

MIN6-K8 cells, seeded at 1 × 10^5^ cells/well in 48-well plates, were pre-incubated for 30 min in Henseleit–Krebs–Ringer buffer (HKRB, 119 mM NaCl, 4.74 mM KCl, 2.54 mM CaCl_2_, 1.19 mM MgCl_2_, 1.19 mM KH_2_PO_4_, 25 mM NaHCO_3_, 10 mM HEPES, pH 7.4) supplemented with 0.2% bovine serum albumin and 2.8 mM glucose. The cells were then incubated for 30 min in HKRB containing 2.8 mM or 16.7 mM glucose, with or without mouse NMU (100 nM) (NMU-23, Peptide Institute, Osaka, Japan) and somatostatin (100 nM) (Peptide Institute). In islet experiments, groups of ten individually size-matched islets were collected for GSIS assays. One hour after pre-incubation with HKRB containing 2.8 mM glucose, islets were incubated for 1 h under the same conditions as for MIN6-K8 cells described above, with or without mouse NMU, NMUR1 agonist 6a (1 μM) (kindly provided by Takayama) [11], human NMU (100 nM) (NMU-25, Peptide Institute), and somatostatin (Peptide Institute). Mouse NMU was used in experiments with mouse islets and MIN6-K8 cells, and human NMU in experiments with human islets. MIN6-K8 cells or islets were pre-incubated with *Bordetella pertussis* toxin (PTX) (100 ng/mL, List Biological Laboratories, Campbell, CA, USA) overnight, and islets were pre-incubated with YM254890 (1 μM, Wako) for 10 min before use. Somatostatin was used as a positive control to suppress GSIS and provide a reliable experimental setting (Fig 1A–1C).

### Short interfering RNA (siRNA) transfection

Mouse Silencer® Select Pre-designed siRNAs s66788 (*Gnai1*), s66789 (*Gnai2*), s66794 (*Gnai3*), and s66800 (*Gnao1*) (Thermo Fisher Scientific, Waltham, MA, USA) were transfected into MIN6-K8 cells using Lipofectamine RNAiMAX Transfection Reagent (Invitrogen, Carlsbad, CA, USA). Control siRNA was Silencer Select–scrambled control siRNA #1 (Thermo Fisher Scientific, Cat. No. 4390843). The cells were treated for 48 h with siRNA or scrambled control siRNA (siSCR), and then subjected to GSIS and measurements of mRNA, mitochondrial membrane potential, and intracellular ATP. In the siRNA knockdown experiments performed to investigate inhibition of signaling and driving effects on insulin secretion (experiments in Fig 1E and 1F), mitochondrial membrane potential (experiment in Fig 4E and 4F), and intracellular ATP (experiment in Fig 1G), we always prepared experimental sets at the same time, in parallel, to confirm the mRNA knockdown efficacy before using the associated findings. In the siRNA knockdown experiments performed to investigate gene expressions related to mitochondrial dynamics (Fig 4A), ER stress (Fig 4B), mitochondrial biogenesis (Fig 4C), and ER calcium channels (Fig 4D), we always confirmed mRNA knockdown efficacy using same samples before investigating other related genes.

### Quantitative RT-PCR (qRT-PCR)

mRNA levels of genes in MIN6-K8 cells were determined 24 h after NMU administration with or without knockdown of *Gnai1*, *Gnai2*, *Gnai3*, or *Gnao1*. Total RNA was purified using the RiboPure Kit (Invitrogen). qRT-PCR was conducted using the TaqMan Fast Universal PCR Master Mix (Thermo Fisher Scientific) on a Thermal Cycler Dice Real-Time System II (Takara Bio, Kusatsu, Japan). Reactions were performed using commercially available primers for the following mouse genes (Thermo Fisher Scientific): *Gnai1*, Mm01165301_m1; *Gnai2*, Mm00492379_g1; *Gnai3*, Mm00802670_m1; *Gnao1*, Mm00494677_m1; *Pgc-1α*, Mm01208835_m1; *Nrf1*, Mm01135606_m1; *Tfam*, Mm00447485_m1; *Mfn1*, Mm00612599_m1; *Mfn2*, Mm00500120_m1; *Fis1*, Mm00481580_m1; *Dnm1l* (*Drp1*), 01342903_m1; *Pink1*, Mm00550827_m1; *Park2*, Mm00450187_m1; *Ddit3* (*Chop*), Mm01135937_g1; *Atp2a3* (sarco/endoplasmic reticulum Ca^2+^-ATPase: SERCA3), Mm00443898_m1; *Ryr2* (ryanodine receptor 2: RyR2), Mm00465877_m1; *Itpr2* (inositol triphosphate receptor 2: IP3R2); and *Gapdh*, Mm99999915_g1. Relative mRNA levels were calculated by normalizing against the level of an internal reference gene (*Gapdh*) in the same sample.

### Measurement of intracellular cAMP

Mice or human islets were pre-incubated for 1 h at 37°C with HKRB containing 2.8 mM glucose and 250 μM PDE inhibitor 3-isobutyl-1-methylxanthine (IBMX, Sigma-Aldrich, St. Louis, MO, USA). Groups of 10 individually size-matched islets were then treated for 1 h with forskolin (10 nM, Sigma-Aldrich) under 16.7 mM glucose, with or without mouse NMU (Peptide Institute), human NMU (Peptide Institute), or somatostatin (Peptide Institute). After incubation, the islets were lysed ultrasonically, and cAMP concentration was measured using the cAMP Biotrak Enzymeimmunoassay system (GE Healthcare, Chicago, IL, USA).

### Measurement of intracellular Ca^2+^ ([Ca^2+^]_i_) with Fura-2

MIN6-K8 cells and dispersed single islet cells were cultured in 35-mm culture plates (Becton Dickinson Labware, Franklin Lakes, NJ, USA). Cells were loaded with 1 μM Fura-2 acetoxymethyl ester (Fura-2-AM, Dojindo, Kumamoto, Japan) in HKRB for 30 min at 37°C. Culture plates were placed on the stage of an integrated fluorescence microscope (BZ-X700, Keyence, Osaka, Japan). Images were captured at 10-s intervals; 340- and 380-nm excitation filters were used for Fura-2-AM dual-wavelength excitation-ratio imaging. For [Ca^2+^]_i_ measurements, the medium was replaced with HKRB containing 2.8 or 16.7 mM glucose with or without mouse NMU (Peptide Institute), ghrelin (Peptide Institute), or somatostatin (Peptide Institute). The fluorescence ratio was recorded for 17 min. At the end of each experiment, cells were exposed to 25 mM KCl for 2 min. Single islet cells exhibiting significant increases in [Ca^2+^]_i_ under 16.7 mM glucose were analyzed as previously reported for β cells [21, 35]. All data were expressed as percent changes relative to average fluorescence ratio in 2.8 mM glucose. Somatostatin was used as a positive control to provide reliable experimental setting (Fig 3C and 3D).

### Analysis of mitochondrial membrane potential

MIN6-K8 cells were cultured in 35-mm culture plates (2 × 10^6^ in 2 mL DMEM) for 20 h after siRNA transfection, and then stimulated with NMU (100 nM) in 16.7 mM glucose for 30 min. They were incubated with 100 nM tetramethylrhodamine ethyl ester (TMRE Mitochondrial Membrane Potential Assay Kit; Abcam, Cambridge, UK) for 20 min at 37°C. Cells were imaged on a BZ-X700 fluorescence microscope. Mitochondrial membrane potential was measured using a bandpass filter with excitation at 549 nm and emission at 575 nm. Short-exposure confocal images were analyzed using ImageJ using the mean fluorescence intensities of arbitrary regions.

### Quantification of ATP content

After siRNA transfection, MIN6-K8 cells were resuspended and seeded at 1 × 10^5^ cells/well in 24-well plates and cultured for 20 h before use. After pre-incubation with 2.8 mM glucose for 30 min, they were incubated for 1 h at 37°C in HKRB buffer containing 2.8 mM or 16.7 mM glucose with or without NMU (100 nM). The cells were then lysed, and ATP content was determined using a colorimetric/fluorometric assay kit (Bio Vision, Milpitas, CA, USA).

### Statistical analyses

Statistical analyses were performed by one-way ANOVA for multiple comparisons, or unpaired *t*-test for comparisons of two mean values. All data are expressed as means ± SEM. *P* < 0.05 was considered statistically significant.

## Results

### NMU suppresses GSIS in both mouse and human islets in a PTX-sensitive manner

To investigate the role of Gα_i/o_ in NMU signaling in pancreatic β cells, we studied GSIS after overnight exposure to PTX. Somatostatin, which suppressed GSIS, was used as a positive control to confirm experimental conditions. NMU suppressed GSIS under high glucose in both MIN6-K8 cells and isolated mouse islets, and PTX abolished NMU-induced suppression of GSIS (Fig 1A and 1B). NMUR1 agonist yielded similar results to those obtained with NMU (Fig 1B). Pre-treatment with the Gαq inhibitor YM254890 had no effect on NMU- or NMUR1 agonist–induced suppression of GSIS (Fig 1C). PTX also abolished NMU-induced suppression of GSIS in isolated human islets (Fig 1D). To determine which subfamily of Gα_i/o_ was responsible for NMUR1 signaling, we studied GSIS after mRNA knockdown of four Gα_i/o_ proteins in MIN6-K8 cells (Fig 1E). Knockdown of *Gnai2* (Gα_i2_ protein) or *Gnao1* (Gα_o_ protein) abolished NMU-induced suppression of GSIS (Fig 1F). Knockdown of *Gnai2* did not affect the mRNA level of *Gnao1*, and vice versa (Fig 1G).

### NMU decreases intracellular cAMP under high glucose

**Fig 2:**
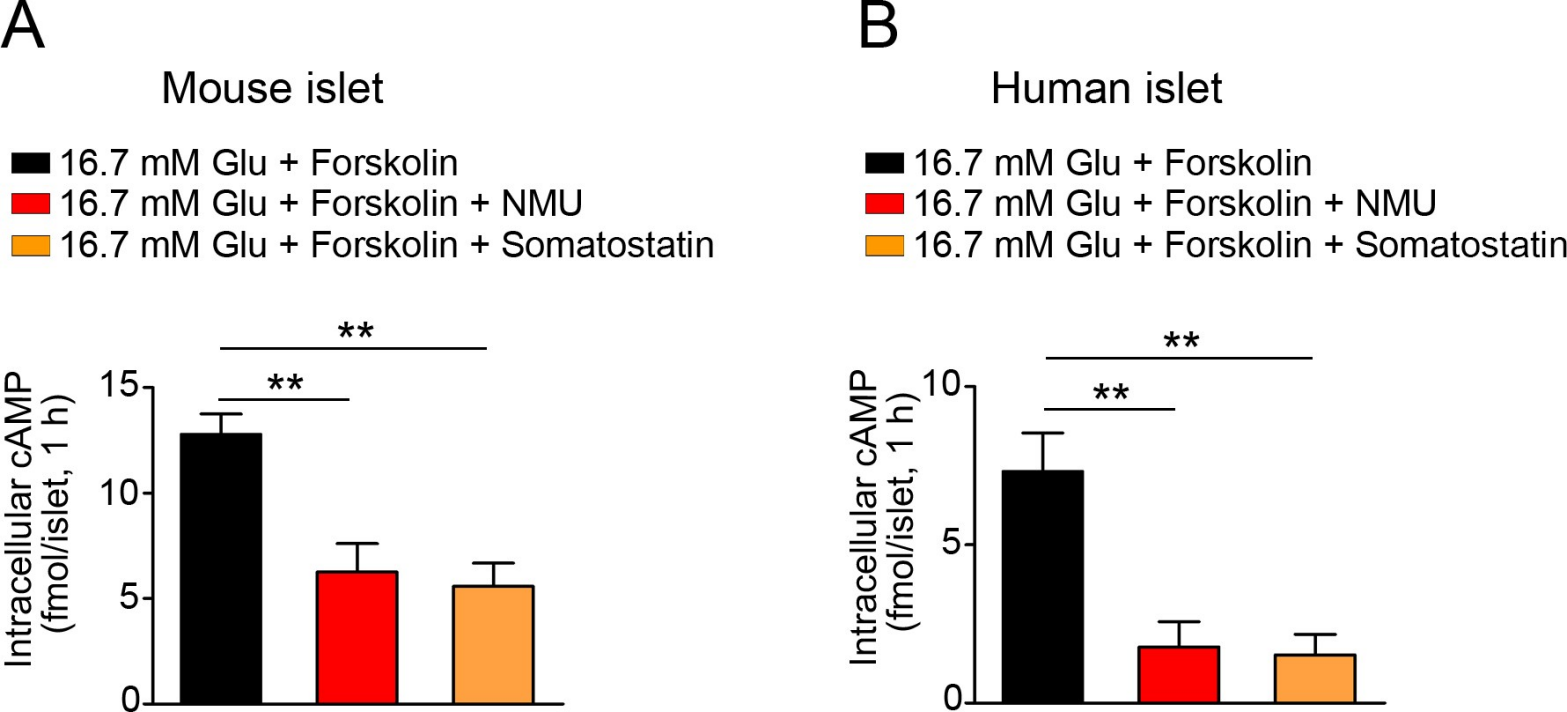
Effects of NMU on intracellular cAMP production. Intracellular cAMP levels in mouse islets (A) (*n* = 4) and human islets (B) (*n* = 4) with or without NMU or somatostatin treatment under 16.7 mM glucose. **P < 0.01.

To determine whether NMU reduces intracellular cAMP, we stimulated intracellular cAMP production in islets with forskolin under high glucose. NMU significantly decreased forskolin-stimulated intracellular cAMP levels in both mouse and human islets (Fig 2A and 2B). Somatostatin also decreased intracellular cAMP levels in islets from both species (Fig 2A and 2B).

### NMU suppresses an increase in [Ca^2+^]_i_ via Gα_i/o_-dependent pathway

**Fig 3:**
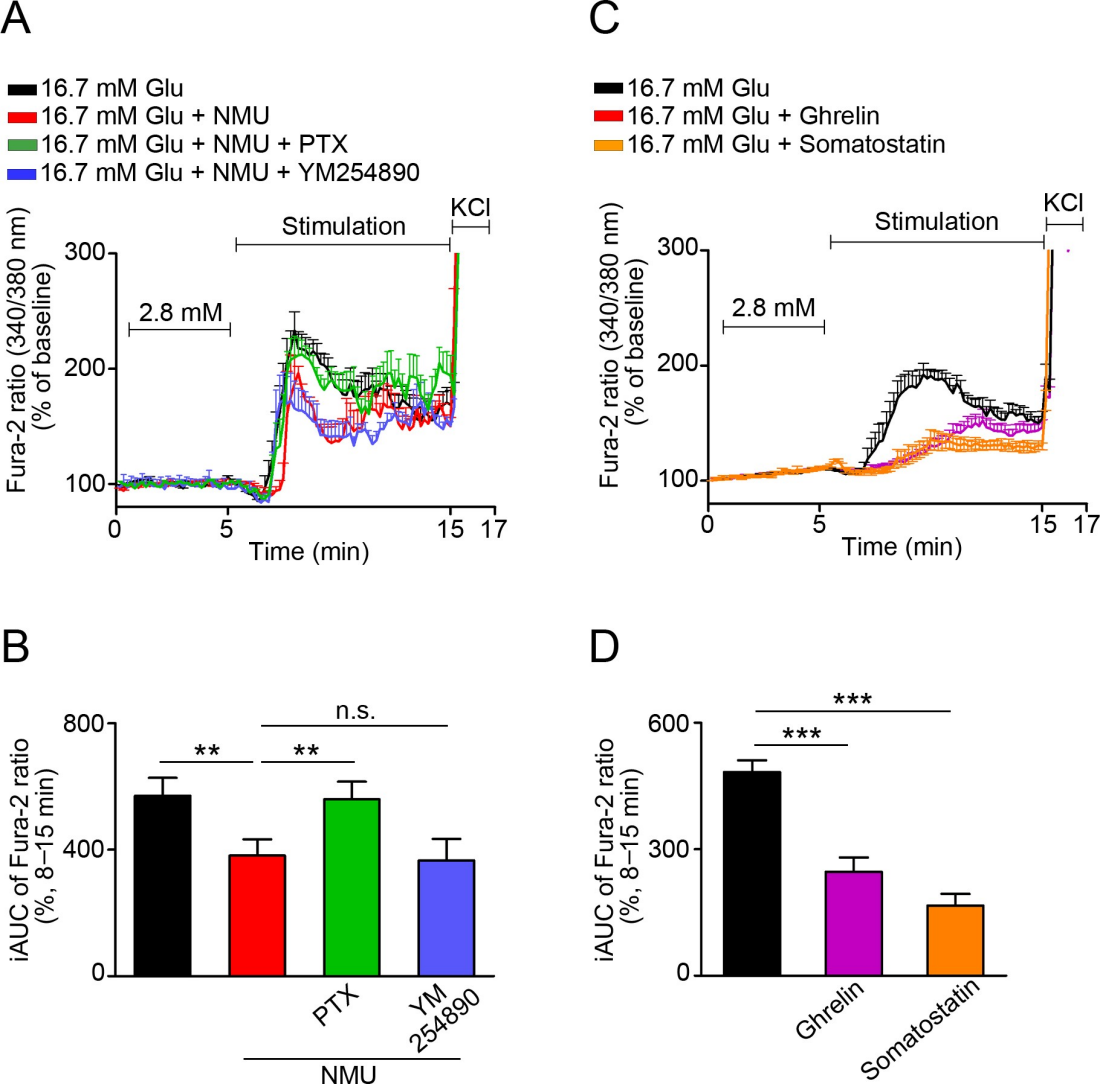
Effects of NMU on [Ca^2+^]_i_ in β cells. (A, C) Representative Fura-2-AM ratios in dispersed mouse β cells in response to NMU with PTX or YM254890 (A), β cell‒derived MIN6-K8 cells in response to ghrelin or somatostatin (C). (B, D) Average incremental AUC (iAUC) (8–15 min) for [Ca^2+^]_i_ in (A) and (C), respectively (*n* = 16). **P < 0.01, ***P < 0.001. n.s., not significant.

NMU suppressed [Ca^2+^]_i_ in dispersed mouse β cells under high glucose; this effect was abolished by pre-treatment with PTX but not YM254890 (Fig 3A and 3B). Both ghrelin and somatostatin also suppressed [Ca^2+^]_i_ under the settings of these experiments (Fig 3C and 3D).

### Knockdown of Gnai2 or Gnao1 restores NMU-induced alterations related to mitochondrial function and ER stress

**Fig 4:**
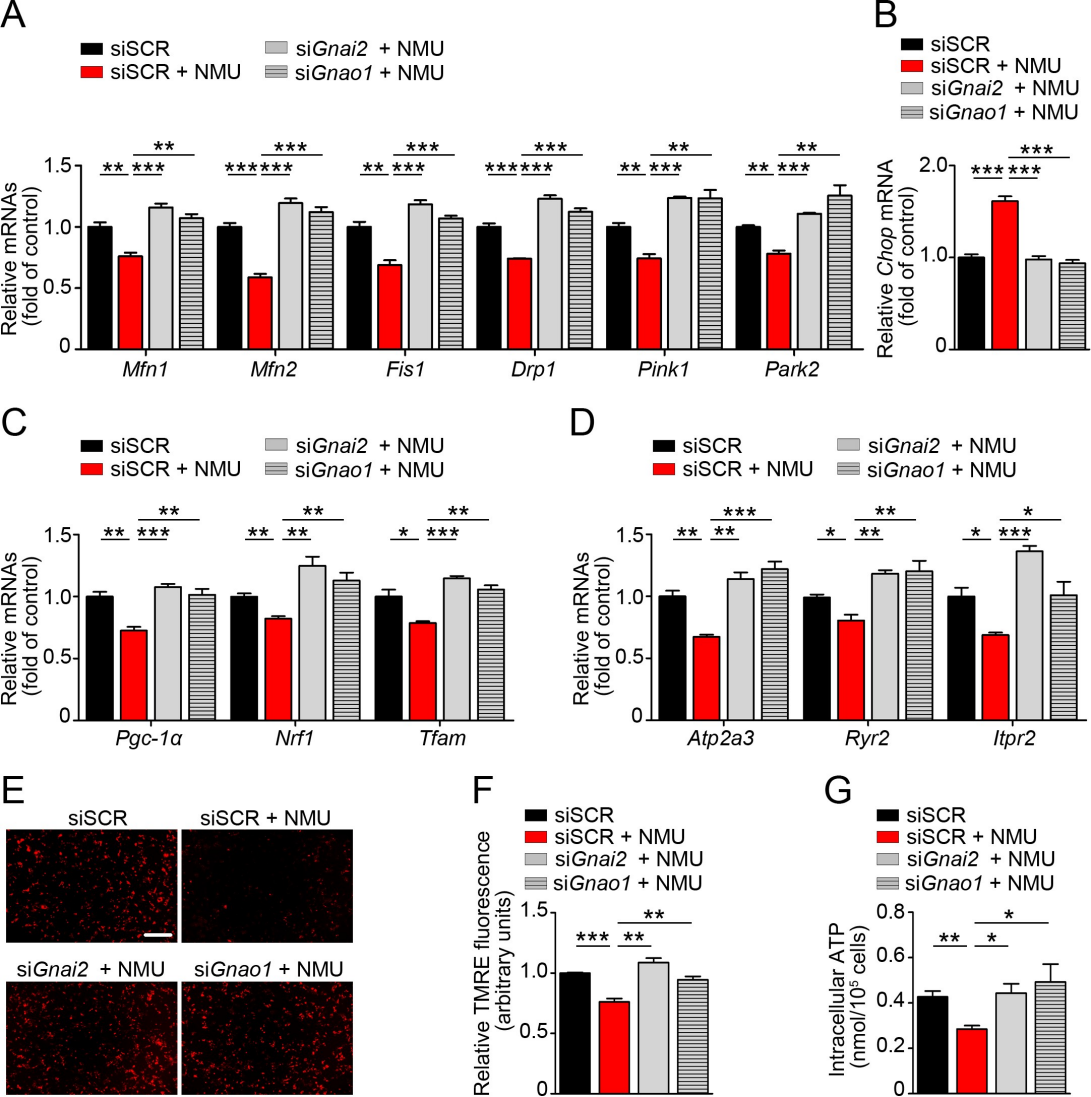
Effects of *Gnai2* and *Gnao1* knockdown on NMU-induced alterations related to mitochondrial function and ER stress in MIN6-K8 cells. (A) mRNAs related to mitochondrial dynamics (n = 4). (B) *Chop* mRNA (n = 4). (C) *Pgc-1α*, *Nrf1*, and *Tfam* mRNAs (n = 4). (D) *Atp2a3*, *Ryr2*, and *Itpr2* mRNAs (n = 4). (E, F) Mitochondrial membrane potential determined by TMRE staining (n = 4). (G) Intracellular ATP level (n = 4). *P < 0.05, **P < 0.01, ***P < 0.001.

Next, we assessed the roles of Gα_i2_ and Gα_o_ in NMU-induced mitochondrial dysfunction and ER stress. In MIN6-K8 cells, NMU significantly decreased the expression of genes related to mitochondrial fusion (*Mfn1*, *Mfn2*), fission (*Fis1*, *Drp1*), and mitophagy (*Pink1*, *Park2*), and expression levels of all of these genes were restored by knockdown of either *Gnai2* or *Gnao1* (Fig 4A). *Gnai2* or *Gnao1* knockdown decreased the expression level of the ER stress marker *Chop*, which was upregulated by NMU administration (Fig 4B). NMU significantly reduced *Pgc-1α*, *Nrf1*, and *Tfam*, genes involved in mitochondrial biogenesis, whereas *Gnai2* or *Gnao1* knockdown significantly upregulated these genes (Fig 4C). Key players in ER Ca^2+^ regulation, including *Atp2a3* (SERCA3), *Ryr2* (RyR2), and *Itpr2* (IP3R2) were downregulated by NMU treatment (Fig 4D). Knockdown of *Gnai2* or *Gnao1* also restored expression of these genes to the same levels as in siSCR-treated samples (Fig 4D). Use of the TMRE probe revealed that administration of NMU to MIN6-K8 cells resulted in a significant reduction of mitochondrial inner membrane potential, suggesting that NMU induced inefficiency in the mitochondrial electron transport chain, whereas *Gnai2* or *Gnao1* knockdown rescued membrane potential (Fig 4E and 4F). NMU significantly decreased glucose-stimulated intracellular ATP level in MIN6-K8 cells; again, this effect was abolished by knockdown of *Gnai2* or *Gnao1* (Fig 4G).

## Discussion

We previously reported that NMU produced in β cells suppresses GSIS and causes β-cell failure via NMUR1 [11, 15]. NMU was upregulated both after chromic palmitate treatment and in diabetic *db/db* mice islets, and then participated in β-cell dysfunction and development of diabetic pathogenesis via induction of mitochondrial dysfunction and ER stress [15]. However, the downstream signal transduction of NMUR1 in β cells has not been elucidated. In this study, we showed that NMUR1 in β cells uses Gα_i/o_ signaling to mediate the detrimental effects of NMU on insulin secretion. Upon NMU binding, NMUR1 preferentially activates Gα_i2_ and Gα_o_, leading to inhibition of intracellular calcium and cAMP level, which in turn suppresses insulin secretion.

The involvement of Gα_i/o_ as an insulinostatic signaling molecule was originally demonstrated by the use of the Gα_i/o_ inhibitor PTX [36, 37]. There are three Gα_i_-encoding genes *Gnai1*, *Gnai2*, and *Gnai3*, which synthesize Gα_i1_, Gα_i2_, and Gα_i3_, respectively, and one Gα_o_-encoding gene *Gnao1*, which synthesizes Gα_o_ [38]. Here, we showed that PTX abolished the suppressive effects of NMU in β cells, whereas knockdown of *Gnai2* and *Gnao1* abolished NMU’s effects, suggesting that Gα_i2_ and Gα_o_ are involved in signal transduction of NMUR1 in β cells. Both Gα_i_ and Gα_o_ belong to the Gα_i/o_ subfamily, and it is usually assumed that if a receptor couples to Gα_i_, it will also couple similarly to Gα_o_ [39]. For example, norepinephrine and somatostatin inhibit insulin release via heterotrimeric Gα_i_ and Gα_o_ proteins by blocking the refilling of the readily releasable granule pool or decreasing granule vesicular docking [40, 41]. In addition, GPCRs can bind to distinct classes of heterotrimeric G-proteins in different cell types. A Gα_s_ and Gα_q_ signaling switch in β cells exposed to chronic hyperglycemia underlies the differential insulinotropic potential of incretins in diabetes [42]. Understanding more about these endogenous G-proteins opens the door to pharmaceutically targeting their activation, which would have major therapeutic potential in diabetes and obesity. Ghrelin activates a Gα_q_-coupled GPCR, growth hormone secretagogue receptor (GHSR), to induce growth hormone secretion in the pituitary [43], whereas in β cells, ghrelin uses Gα_i2_ to attenuate glucose-induced Ca^2+^ signaling and insulin secretion [21]. Ghrelin activates GHS-R1a in pancreatic β cells, initiating the Gα_i/o_ signaling pathway via heterodimer formation with somatostatin receptor 5 [21, 44]. Despite the fact that NMUR1 and NMUR2 were discovered as GHSR homologs [45], it remains to be determined whether such dimerization occurs between NMUR1 and somatostatin receptors. Although the mechanisms underlying the involvement of Gα_i_ and Gα_o_ signaling in NMU inhibitory effects on insulin secretion require detailed investigation, including studies at the structural level, our findings provide important leads regarding the endogenous NMUR1 transduction pathway. In addition, the diversity of GPCR coupling to various types of G-proteins and the subsequent activation of distinct intracellular signal transduction may be explained by alterations in guanine nucleotide exchange factor or Gα subunit–regulated GTP hydrolysis [46, 47].

NMUR1 activates Gα_q_ to increase intracellular Ca^2+^ levels in various cell types [24, 48]; however, in neurons of mouse dorsal root ganglia and hippocampus, NMUR1 binds to Gα_i/o_ to inhibit T-type and L-type Ca^2+^ channels, respectively [30, 31]. In this study, PTX abolished NMU-suppressed calcium influx in β cells, suggesting that using different Gα proteins, NMUR1 apparently exhibits opposite effects on calcium mobilization, depending on different cell types.

The other insulin-releasing signal in β cells is intracellular cAMP [49, 50]. The intracellular cAMP level is determined by a balance between production by adenylyl cyclases and degradation by cyclic nucleotide phosphodiesterase (PDE). Here, we showed that NMU suppresses forskolin-stimulated cAMP in mouse and human islets in the presence of the PDE inhibitor IBMX, suggesting that NMU affects cAMP levels by regulating initiation of cAMP synthesis rather than degradation. Thus, NMU may suppress GSIS at least partly by decreasing the intracellular cAMP level.

Previously, we showed that NMU induced β-cell failure by triggering mitochondrial dysfunction and ER stress [15]. In this study, we showed that knockdown of Gα_i2_ and Gα_o_ in β cells restored NMU-induced mitochondrial dysfunction and ER stress. These findings suggest that Gα_i2_ and Gα_o_ proteins couple to NMUR1 and induce β-cell dysfunction. In addition, Gα_i2_ and Gα_o_ proteins are crucial in the NMU–NMUR1 regulating glucose-stimulated Ca^2+^ signaling and insulin secretion in β cells. Knowledge of the NMU signaling cascades, NMU–NMUR1–Gα_i2_ and Gα_o_–cAMP–calcium–insulin, may provide insight into β-cell biology and the pathogenesis of diabetes related to NMU.
